# Structural evolution of nanoscale metallic glasses during high-pressure torsion: A molecular dynamics analysis

**DOI:** 10.1038/srep36627

**Published:** 2016-11-07

**Authors:** S. D. Feng, W. Jiao, Q. Jing, L. Qi, S. P. Pan, G. Li, M. Z. Ma, W. H. Wang, R. P. Liu

**Affiliations:** 1 State Key Laboratory of Metastable Materials Science and Technology, Yanshan University, Qinhuangdao, 066004, China; 2 Institute of Physics, Chinese Academy of Sciences, Beijing, 100190, China; 3 College of Materials Science and Engineering, Taiyuan University of Technology, Taiyuan, 030024, China; 4 Department of Materials Science and Engineering, The University of Tennessee, Knoxville, Tennessee, 37996, USA

## Abstract

Structural evolution in nanoscale Cu_50_Zr_50_ metallic glasses during high-pressure torsion is investigated using molecular dynamics simulations. Results show that the strong cooperation of shear transformations can be realized by high-pressure torsion in nanoscale Cu_50_Zr_50_ metallic glasses at room temperature. It is further shown that high-pressure torsion could prompt atoms to possess lower five-fold symmetries and higher potential energies, making them more likely to participate in shear transformations. Meanwhile, a higher torsion period leads to a greater degree of forced cooperative flow. And the pronounced forced cooperative flow at room temperature under high-pressure torsion permits the study of the shear transformation, its activation and characteristics, and its relationship to the deformations behaviors. This research not only provides an important platform for probing the atomic-level understanding of the fundamental mechanisms of high-pressure torsion in metallic glasses, but also leads to higher stresses and homogeneous flow near lower temperatures which is impossible previously.

Metallic glasses (MGs) show unique mechanical properties such as high strength and elastic limit compared to their crystalline counterparts^1^,^2^,^3^,^4^. However, their applications are limited by failing under one dominant shear load when also under uniaxial load at room temperature^5^,^6^. Rodney and Schuh have addressed that the current challenges in this field are both technological, improving the mechanical reliability of MGs, and fundamental, developing a consistent theory of plastic deformation similar to the dislocation theory^7^. Inspiringly, MGs show enhanced strength, ductility, and fracture toughness when shrunk to the nanoscale^8^,^9^. In fact, the intrinsic mechanical length scale in MGs is associated with clusters of about 100 atoms (*i.e*., a few atomic diameters in size) that serve as shear transformation zones (STZs) upon deformation^10^,^11^. It has been shown that an initial STZ may trigger a cascade of rearrangements during deformation^12^. At yield stress, intrinsically, the STZs coalesce and assemble into large and nano-scaled thin planar bands, generally called shear bands^13^. For example, Sha *et al*. concluded that when the size of the STZ aggregates accumulating at the MG surface reaches a critical value comparable to the shear band width, shear band initiation can take place^14^. Shear band formation and propagation is a highly localized and nano-scale deformation mechanism in MGs strained at room temperature^15^,^16^,^17^. Our previous work that considers the “liquid-like” character of shear bands provided important details that extend earlier studies on shear bands^18^. The deformation behavior limits the structural reliability of MGs. When test temperature approached the glass transition temperature, there is an inhomogeneous-to-homogeneous flow transition, which can be described by the STZ model^19^,^20^,^21^,^22^. Fan *et al*. found that subnano-scale rearrangements of a small number of atoms could trigger thermally activated deformation, and activation barrier, i.e. activation energy, is independent of temperature^23^. It is worth noting that shear bands even occur at temperature close to glass transition temperature because of high strain-rate^24^. So, large and homogenous plastic strain in MGs at lower temperatures and higher stresses is hard to realise^24^,^25^. To alleviate this problem, grasping how STZs coalesce into shear bands is very necessary, thereby controlling their assembling manner.

High-pressure torsion (HPT) is a popular method applying a complex stress state to achieve large plastic strain, where the sample is subjected to a compressive applied pressure and a torque simultaneously, and is used to fabricate ultrafine grained crystalline materials^26^,^27^. For example, Révész *et al*.^28^ found that there are differences in the sign of the atomic distortion obtained in direction parallel to the sample surface and along the cross section, indicating strong structural anisotropy in MGs by HPT at room temperature. This indicates that HPT can be applied to activate microscopic deformation carriers and achieve large strain therein in MGs. Intensive work has also shown that HPT processing could promote structural rejuvenation in MGs and lead to homogenous deformation^29^,^30^,^31^. For example, using high-energy X-ray diffractio n, Dmowski *et al*.^29^ addressed the changes in the pair distribution functions by the HPT technique, suggesting structural rejuvenation by heavy deformation. Meng *et al*. found that HPT could make the deformation mode change from heter ogeneous, localized deformation to homogeneous deformation in Zr_50_Cu_40_Al_10_ MGs^30^. Adachi *et al*. pointed that HPT could induce structural defects, which overcomes the compositional limitations and improves the mechanical properties of MGs^31^. These pioneering studies, all aimed at understanding the HPT, had a significant impact. Nevertheless, how these shear transformations coalesce and assemble into shear bands, the correlation between the characteristics of the shear transformations and the atomic structure, and the energy distribution under HPT remain elusive, thereby making it difficult to study the shear transformation, its activation and c haracteristics, and its relationship to the deformations behaviors under HPT.

In this study, we analysed the evolution of shear transformations aggregation under HPT at room temperature in nanoscale Cu_50_Zr_50_ MGs through molecular dynamics (MD) simulations. The MD simulations are limited to high cooling rates and small sizes but provide the mechanistic observations needed to relate the shear transformations and HPT deformation in nanoscale MGs^32^. It is shown that shear bands can be suppressed and constrained by HPT in nanoscale Cu_50_Zr_50_ MGs, resulting in forced cooperative flow. It is worth noting that multiple and extended shear band formation in MGs during HPT has already been shown in the experimental work^33^. Contrary to this, we clearly demonstrated that the nanoscale MGs during HPT can lead to homogeneous flow, which may be cooling rate and size differences between the model and experiments (see Figs S1 and S2 in Supporting information). And abundant atomic-scale shear transformations cooperate strongly with each other in the nanoscale MGs under HPT. The atoms possess lower five-fold symmetries and higher potential energies during HPT, making them more likely to participate in shear transformations. Factors leading to these novel deformation mechanisms in nanoscale MGs under HPT are discussed, and the effect of torsion speed on the deformation behaviour of nanoscale MGs was also considered.

## Results and Discussion

### Nanoscale Cu_50_Zr_50_ MGs and Cu_50_Zr_50_ crystals under HPT

**Figure 1:**
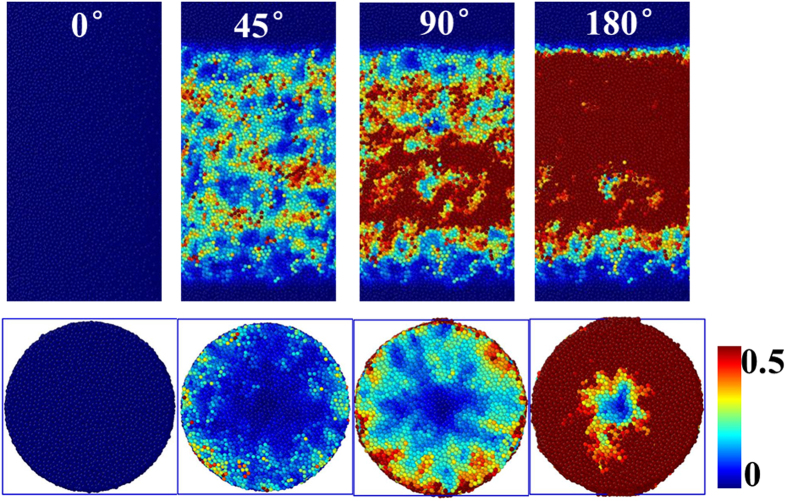
A sequence of surface images (up) and cross-sectional images (down) that demonstrate deformation in nanoscale Cu_50_Zr_50_ MGs under HPT.

Figure 1 shows a sequence of images that demonstrate deformation in nanoscale Cu_50_Zr_50_ MGs under HPT. The deformation process was monitored by using the so-called local atomic shear strain *η*_*i*_^*Mises*^, referenced to the relaxed glass prior to loading^34^. The presence of regions with relatively large local atomic shear strain indicates a high density of shear transformations^35^, which represents the collective and inelastic shearing of atoms in response to an applied shear stress^36^,^37^. With increasing torsion angle (TA), the number of atoms participating in the deformation gradually increased. Shear translations were distributed across most of the outer surface of the MGs at the initial stages of deformation, such as TA = 45°. With increasing TA the whole of the outside surface turned red (see Movie S1 in Supplementary materials). Meanwhile, in accordance with the radial dependent shear deformation, the MG exhibited strain gradient, *i.e*., when the TAs were identical, the local atomic shear strain *η*_*i*_^*Mises*^ of atoms located at a larger radius were greater. With the increase of TA, local atomic shear strain *η*_*i*_^*Mises*^ of atoms located near the center also increased (see Movie S2 in Supplementary materials). The above meant that the atoms were gradually involved in shear transformations under HPT deformation. Shear transformations could pervade themselves into the whole effective area in the MG, which implied that the HPT in MGs may realise large continuous deformation. Shear transformations may have strong dependence on the preparation history of the sample. Our previous work proved that higher initial quenching temperatures or cooling rates decrease short range order and clusters in MGs^38^. Smaller clusters prevail in liquids at higher initial quenching temperature, and are passed into the glass by fast quenching. Under such conditions, shear transformations will be easier to cooperate strongly with each other, and propagate throughout the whole MG. Besides the preparation history of the sample, the size of MGs makes the deformation mode change from localized deformation to homogeneous deformation when the relevant size is under a critical value, which is interpreted in terms of competition between heterogeneous shear band and homogeneous cooperative flow^39^.

**Figure 2:**
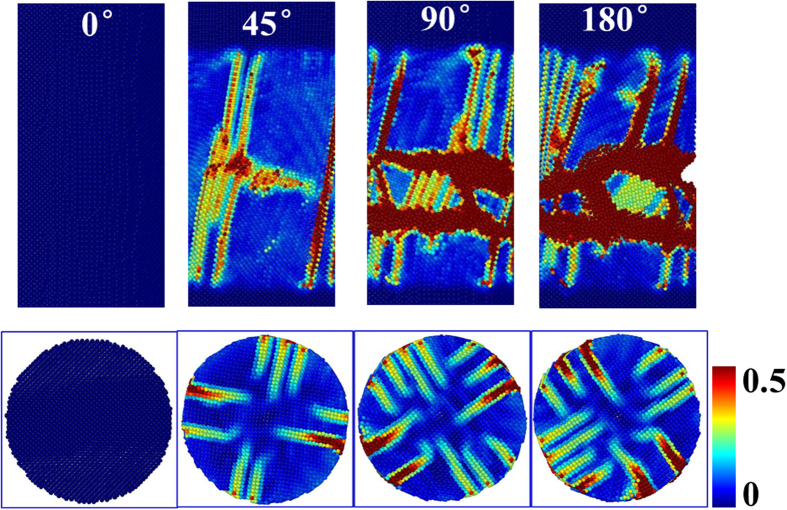
A sequence of surface images (up) and cross-sectional images (down) that demonstrate deformation in nanoscale Cu_50_Zr_50_ crystals under HPT.

For comparison purposes, Cu_50_Zr_50_ crystal loading was performed under the same conditions as used on the Cu_50_Zr_50_ MG in the present work, as shown in Fig. 2. Through-bands began to form on the outer surface of the models at the initial deformation. With increasing TA, bands gradually increased in number, but there was no evidence that almost all atoms were involved in the deformation as was seen in MGs (see Movie S3 in Supplementary materials). In other words, a few parts of the crystal structure were involved in the deformation. This may be due to the crystalline structure resulted in non-uniform properties at the length-scale of their microstructure.

The above results showed that the evolution of localised shear strain in nanoscale Cu_50_Zr_50_ MG under HPT was distinctly different from that in nanoscale Cu_50_Zr_50_ crystals. At an atomic scale, accommodation of shear strain in nanoscale MGs under an applied stress was believed to occur by local rearrangement of atoms around free volume regions, unlike motion in crystalline alloys. With increasing TA, several embryonic shear transformations regions in the nanoscale MG merged and kept growing. However, the size of the shear transformations aggregates accumulating in the nanoscale MG during HPT does not reach a critical value comparable to the shear band width, so no apparent shear band formation was observed, which suggested that HPT may facilitate forced cooperative flow by activation of numerous diffuse shear transformations for nanoscale MGs. Nanoscale MGs effectively exhibited forced cooperative flow consistent with typical superplastic behaviour under HPT. In this case, not only did a large number of distributed shear transformations nucleate and remain active as deformation progressed, but new shear transformations appeared, creating an increasingly dense shear transformation network. The driving force for shear band propagation was the stored elastic energy^40^. For nanoscale MGs under HPT, the stored energy was dissipated into the activation and formation of a large number of homogeneously distributed shear transformations rather than into a localised single shear band as seen under uniaxial compression. The homogeneous energy release was not sufficient to drive shear band propagation. Consequently, nanoscale MG under HPT deformed homogeneously, which didn’t exhibit localised deformation by one major shear band propagation event.

### HPT and uniaxial compression of nanoscale Cu_50_Zr_50_ MGs

**Figure 3:**
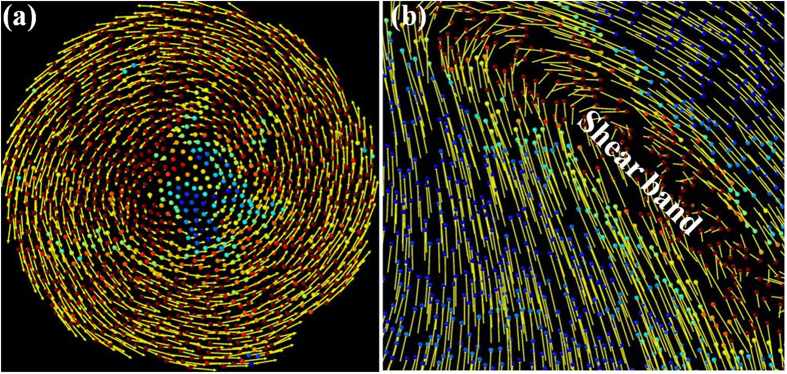
Atomic displacement vectors and local shear strain under (**a**) HPT and (**b**) uniaxial compression in nanoscale Cu_50_Zr_50_ MGs. The yellow arrows represent the atomic displacement vectors, and atoms are coloured according to their local shear strain.

We were particularly interested in shear band formation mechanisms. In order to understand the deformation behaviour better, in the following section, the change of displacement at atomic level is shown in detail. As shown in Fig. 3(a), atomic displacement vectors show that atoms rotated in an anticlockwise manner on the whole under HPT: the displacement of atoms located in the outer ring was large, while the displacement of atoms located near the center was small. On the whole, all atoms moved in a regular fashion. Figure 3(b) shows that atoms of Cu_50_Zr_50_ MG in the shear band moved desultorily compared with those in the matrix under uniaxial compression. These atoms interacted with each other and were irregular, which resulted in instability during the deformation process. The movement of all atoms under HPT is relatively regular, and this suggests that the HPT deformation of MGs was stable. Under applied HPT, shear transformations could multiply and pervade the whole effective area in the MG, which renders the shear transformations unable to develop a mature shear band, resulting in more stable shear deformation.

The process of shear band development comprises shear band initiation and shear band propagation. The size of the shear transformations aggregates accumulating in the MG under uniaxial compression reach the critical value comparable to the shear band width, so a mature shear band was observed. However, during HPT deformation, shear band initiation could not occur as the size of shear transformation aggregates accumulating at the MG surface didn’t reach a critical value comparable to the shear band width; on the other hand, shear band propagation was also blocked because of the atomic shear transformations. This may have important implications for improving the deformation behaviour of MGs by adjusting shear band formation.

### Quantifying the shear transformations

**Figure 4:**
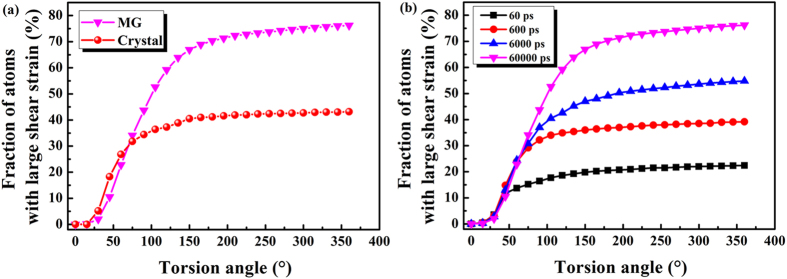
(**a**) The fraction of atoms with relatively large atomic shear strain in MGs and crystals as a function of torsion angle; (**b**) The fraction of atoms with relatively large atomic shear strain in MGs as a function of torsion angle for various torsion periods.

As the evolution of shear transformations is characterised by atomic shear strain during HPT, the fraction of atoms with relatively large atomic shear strain (*i.e*., STZs) during HPT is shown in Fig. 4. Atoms with a higher than 20% local shear strain were considered to achieve large shear deformation^41^. Thus, HPT deformation can be quantified as the ratio of the number of atoms in the large shear deformation region relative to the total number of atoms in the sample. Figure 4(a) shows the fraction of atoms with relatively large atomic shear strain, *η*_*i*_^*Mises*^ > 0.2, as a function of TA. It was found that, when TA = 360°, their fraction in MG was 76.2%, while that in crystal was 43.2%. During deformation, the fraction of atoms undergoing large shear deformation in MG reached 76.2%, implying that a substantial fraction of the material experienced structural changes that facilitated shear transformations. This quantitatively illustrated that the MGs experienced shear transformations, while only to a partial extent, atoms of crystals were involved in HPT deformation. At the same time, the MG reached a plateau at TA = 150°, while the crystals reached their plateau at TA = 75°. This suggested that the MG can resist the damage induced by HPT more effectively in the longer-term. MG, under HPT, underwent a substantial fraction of structural changes that facilitated shear transformations which, in turn, gave rise to the observed superplastic-like behaviour.

To understand, or predict, the HPT deformation of MGs better, it is important to make a qualitative investigation of the effects of torsion period, *e.g*., torsion speed. The results demonstrated the significant influence of torsion period on the deformation behaviour of MGs. Figure 4(b) shows the fraction of atoms with relatively large atomic shear strain as a function of TA for various torsion periods. It can be seen that the accumulation rate of shear transformations was sensitive to torsion period. With increasing torsion period, the shear transformations accumulated in abundance because of the adequate duration allowing this to come about. The effect of torsion period on the HPT deformation of MGs suggested that a higher torsion period led to a greater extent of shear transformations. The results indicated that the activation of a high density of shear transformations can readily lead to the percolation of shear transformations or macroscopic plastic deformation in MGs.

### The “liquid-like” character during HPT

**Figure 5:**
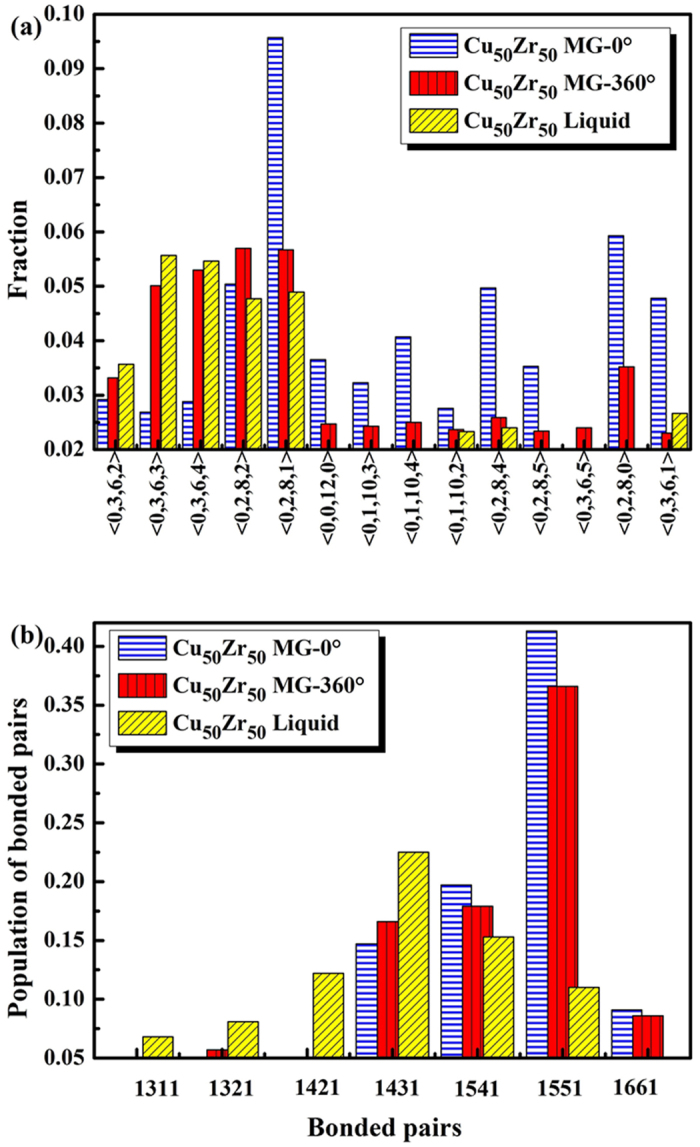
(**a**) The distributions of the top 14 Voronoi polyhedra of the Cu_50_Zr_50_ MG at TA (torsion angle) = 0° and 360°, and Cu_50_Zr_50_ liquid; (**b**) Comparison of bonded pairs in the three aforementioned models.

Figure 5(a) shows the distributions of the top 14 Voronoi polyhedra (VP) of the Cu_50_Zr_50_ MG at TA = 0° and TA  = 360°, and Cu_50_Zr_50_ liquid, where the element types were not distinguished. In the Voronoi tessellation method^42^, the simulation cell is divided into VP around each atom. The polyhedra can be characterised by the Voronoi index [*n*_3_, *n*_4_, *n*_5_, *n*_6_], where *n*_*i*_ denotes the number of *i*-edged faces of the VP. An *i*-edged face reflects the local symmetry of the central atom with some nearest-neighbour atoms in a certain direction. The distributions of Cu_50_Zr_50_ MG (TA = 0°) were mainly located in the side of pentagon dominant VP, such as <0,2,8,1> and <0,2,8,4>. While the distributions of Cu_50_Zr_50_ MG (TA = 360°) showed a close resemblance to that of the corresponding Cu_50_Zr_50_ liquid. They were located in the side of VP, where pentagon accounted for a minor proportion of the total, and <0,2,8,1> VP were regarded as the watershed thereof. This suggested that HPT destroyed the short-range order structure, in which fivefold symmetry was dominant.

To quantify free volume change during HPT deformation, the content and corresponding volumes of the top 14 Voronoi polyhedra (VP) were calculated. To be more comparable with this approach, a parameter^43^ was proposed such that:


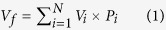


where *V*_*f*_ denotes the effective free volume, *N* denotes the total number of types of VP, *V*_*i*_ is the volume of VP *i*, and *P*_*i*_ is the content of VP *i*. The *V*_*f*_ of Cu_50_Zr_50_ MG at T = 360° (16.76) was greater than that of Cu_50_Zr_50_ MG at T = 0° (15.82), and was close to the *V*_*f*_ of Cu_50_Zr_50_ liquid (17.91). Figure 5(b) shows the comparison of bonded pairs. A bonded pairs index with four integers *ijkl* was raised by Honeycutt and Andersen (H-A)^44^. The first number *i* in the H-A index is used to identify the bonding of two given atoms (*i* = 1 for bonded pairs and *i* = 2 for non-bonding atoms); *j* is the number of nearest neighbors shared jointly by the two atoms; and *k* is the number of bonds among the shared neighbors. The fourth digit *l* is needed in case the first three numbers are the same but the bond geometries are different. To analyse further that information contained in the bonded pairs, Pan^45^ divided the bonded pairs into two categories: when the shared neighbours connected to form a ring, *i.e*. when *k* = *j*, they were designated “saturated bonded pairs”; otherwise, they were designated “unsaturated bonded pairs”. The degree of saturation of the system, *I*, is defined as:


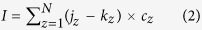


where *j*_*z*_ is index *j* of bonded pairs *z*, and *k*_*z*_ is index *k* of bonded pairs *z*. Here *c*_*z*_ is the proportion of bonded pairs *z*. The greater *I* represents the more defects. And when MGs have no defect (ideal MGs), the I is 0. In contrast with Cu_50_Zr_50_ MG at TA = 0°, 1551 saturated bonded pairs of Cu_50_Zr_50_ MG, at TA = 360°, were destroyed. Through statistical calculations, the unsaturated index of the Cu_50_Zr_50_ MG at TA = 0° was found to have been 0.344, while that of the Cu_50_Zr_50_ MG at TA = 360° was 0.504. Note that the unsaturated index of Cu_50_Zr_50_ MG at TA = 360° was more similar to that (0.839) found in the Cu_50_Zr_50_ liquid. It also verified the fact that the HPT destroyed some five-fold symmetry in MG, rendering it easier to experience shear transformations. Five-fold symmetry is associated with dense packing in amorphous materials^46^. So the destruction of five-fold symmetry reduced dense packing, and increased the free space within which atoms can move, a result which was consistent with the expansion of the effective free space *V*_*e*_ in Cu_50_Zr_50_ MG at T = 360°. These suggested that HPT made the atoms in the MGs more homogeneous and more akin the liquid state, providing MGs with effective free volume so that they could experience shear transformations.

### The potential energies during HPT

**Figure 6:**
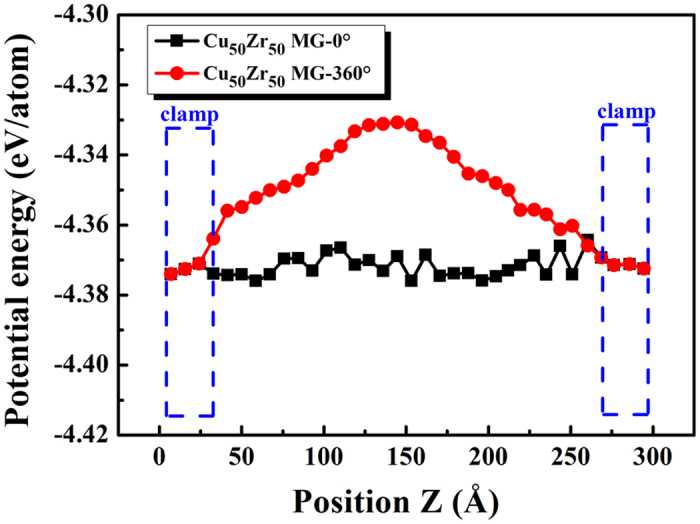
Change in potential energies of atoms of the Cu_50_Zr_50_ MG at TA = 0° and 360°.

The observed HPT-induced emergence of shear transformations in MGs can be rationalised in terms of energetics. To reveal physical mechanisms of deformation as a function of energetics, the potential energy at TA = 0° and 360° was calculated, as shown in Fig. 6. It shows that the potential energy at TA =  360° was higher than that at TA = 0° except for those located within the clamps. HPT made atoms have higher potential energies, which increased their probability of participating in plastic deformation by way of a shear transformation because the energy cost of moving such atoms was lowered, akin to the free volume driven mechanism for homogeneous flow. This implies that the structure of the deformed volume becomes effectively rejuvenated by the HPT^29^. It was reasonable to conclude that the HPT stress state, with its greater free volume and higher potential energy per atom, may suppress catastrophic shear band formation and explain the simulated observations presented here. The shear transformation involved a localised cluster of atoms that underwent intense distortion from an initial to final equilibrium position through an intermediate activated state of high-energy and large-volume^47^. In other words, if enough energy were applied to activate a sufficiently high density of shear transformations, then homogeneous plastic deformation can occur in any MGs at room temperature depending on the activation, density, and percolation mode of the shear transformations.

## Conclusion

In summary, we have performed a series of MD simulations of HPT in nanoscale MGs. It is found that the nanoscale MGs experienced forced cooperative flow. In this process, shear transformations under applied HPT stress could multiply and pervade the whole effective area in the MG, meaning that there is no obviously stress concentration. Therefore shear transformations cooperate and homogenously flow could be realized. Nanoscale MGs, compared to their crystalline counterparts, conferred significant performance advantages under HPT. We demonstrate the effect of torsion speed on the deformation behaviour of nanoscale MGs, with results suggesting that a higher torsion period led to a greater degree of forced cooperative flow. Our results show that HPT could prompt atoms to possess lower five-fold symmetries, higher potential energies and larger volume, making them more likely to participate in shear transformations.

## Methods

The simulations were performed using the embedded atom method (EAM) potential supplied in large-scale atomic/molecular massively parallel simulator (LAMMPS)^48^,^49^. The dimensions of the Cu_50_Zr_50_ MGs model system used in the calculations were 15.5 × 15.5 × 30 nm in the *x*-, *y*-, and *z*-directions, respectively, containing approximately 420,000 atoms. It was melted and equilibrated with periodic boundary conditions for 1 ns at 2000 K, which is above the melting point of these alloys. The model was then cooled at an MD cooling rate of 0.5 K ps^−1^ to a glass state (300 K). As a comparison, the B2 Cu_50_Zr_50_ crystal model, containing approximately 420,000 atoms, was also built and relaxed with periodic boundary conditions at 300 K for 5 ns to ensure the energy minimization of the model. The NPT ensemble (constant number, constant pressure, and constant temperature) was used for these processes, with the temperature controlled by a Nose-Hoover thermostat and the pressure controlled at zero using a Nose-Hoover barostat.

The cylindrical-shaped nanoscale MG and crystal models are prepared by cutting, 15 nm in diameter and 30 nm in the z-axis. With 1 GPa applied pressure in the z-direction, the models were relaxed for 3 ns at 300 K. Thereafter, these models were simulated by rotating the rigid atoms at one end (3 nm in thickness) along the z-axis, while keeping the rigid atoms at the other end (3 nm in thickness) unchanged, being akin to the clamps. Free surfaces were imposed in the x- and y- directions during the torsion. Torsion speeds were 1/60 revolution/ps, 1/600 revolution/ps, 1/6000 revolution/ps, and 1/60000 revolution/ps, respectively.

## Additional Information

**How to cite this article**: Feng, S. D. *et al*. Structural evolution of nanoscale metallic glasses during high-pressure torsion: A molecular dynamics analysis. *Sci. Rep.* **6**, 36627; doi: 10.1038/srep36627 (2016).

**Publisher’s note:** Springer Nature remains neutral with regard to jurisdictional claims in published maps and institutional affiliations.

## Supplementary Material

Supplementary Movie S1

Supplementary Movie S2

Supplementary Movie S3

Supplementary Materials

## References

[b1] Greer A. L., Cheng Y. Q. & Ma E. Shear bands in metallic glasses. Mater. Sci. Eng. R 74, 71–132 (2013).

[b2] Hufnagel T. C., Schuh C. A. & Falk M. L. Deformation of metallic glasses: Recent developments in theory, simulations, and experiments. Acta Mater. 109, 375–393 (2016).

[b3] Cheng Y. Q. & Ma E. Atomic-level structure and structure–property relationship in metallic glasses. Prog. Mater. Sci. 56, 379–473 (2011).

[b4] Huang E. W. . Microyielding of core-shell crystal dendrites in a bulk-metallic-glass matrix composite. Sci. Rep. 4, 4394 (2014). 24637714 10.1038/srep04394 PMC3957129

[b5] Chen S. H. . Loading-rate-independent delay of catastrophic avalanches in a bulk metallic glass. Sci. Rep. 6, 21967 (2016). 26912191 10.1038/srep21967 PMC4766412

[b6] Wu F. F., Chan K. C., Jiang S. S., Chen S. H. & Wang G. Bulk metallic glass composite with good tensile ductility, high strength and large elastic strain limit. Sci. Rep. 4, 5302 (2014). 24931632 10.1038/srep05302 PMC4058877

[b7] Rodney D. & Schuh C. Distribution of thermally activated plastic events in a flowing glass. Phys. Rev. Lett. 102, 235503 (2009). 19658948 10.1103/PhysRevLett.102.235503

[b8] Greer J. R. & De Hosson J. T. M. Plasticity in small-sized metallic systems: Intrinsic versus extrinsic size effect. Prog. Mater. Sci. 56, 654–724 (2011).

[b9] Chen D. Z. . Nanometallic glasses: size reduction brings ductility, surface state drives its extent. Nano Lett. 13, 4462–4468 (2013). 23978318 10.1021/nl402384r

[b10] Qiao J. & Pelletier J. Dynamic mechanical relaxation in bulk metallic glasses: a review. J. Mater. Sci. Technol. 30, 523–545 (2014).

[b11] Falk M. L. & Langer J. S. Dynamics of viscoplastic deformation in amorphous solids. Phys. Rev. E 57, 7192–7205 (1998).

[b12] Demkowicz M. J. & Argon A. S. Autocatalytic avalanches of unit inelastic shearing events are the mechanism of plastic deformation in amorphous silicon. Phys. Rev. B 72, 245206 (2005).

[b13] Schuh C. A. & Lund A. C. Atomistic basis for the plastic yield criterion of metallic glass. Nat. Mater. 2, 449–452 (2003). 12792648 10.1038/nmat918

[b14] Sha Z., Qu S., Liu Z., Wang T. & Gao H. Cyclic deformation in metallic glasses. Nano Lett. 15, 7010–7015 (2015). 26422317 10.1021/acs.nanolett.5b03045

[b15] Sha Z. D., Pei Q. X., Liu Z. S., Zhang Y. W. & Wang T. J. Necking and notch strengthening in metallic glass with symmetric sharp-and-deep notches. Sci. Rep. 5, 10797 (2015). 26022224 10.1038/srep10797 PMC4448266

[b16] Zhou H. . Non-localized deformation in metallic alloys with amorphous structure. Acta Mater. 68, 32–41 (2014).

[b17] Li Q.-K. & Li M. Atomic scale characterization of shear bands in an amorphous metal. Appl. Phys. Lett. 88, 241903 (2006).

[b18] Feng S. . Atomic structure of shear bands in Cu_64_Zr_36_ metallic glasses studied by molecular dynamics simulations. Acta Mater. 95, 236–243 (2015).

[b19] Schuh C. A., Lund A. C. & Nieh T. G. New regime of homogeneous flow in the deformation map of metallic glasses: elevated temperature nanoindentation experiments and mechanistic modeling. Acta Mater. 52, 5879–5891 (2004).

[b20] Yang B., Wadsworth J. & Nieh T. G. Thermal activation in Au-based bulk metallic glass characterized by high-temperature nanoindentation. Appl. Phys. Lett. 90, 061911 (2007).

[b21] Qiao J. W. . Low-temperature shear banding for a Cu-based bulk-metallic glass. Scripta Mater. 63, 871–874 (2010).

[b22] Song S. X., Jang J. S. C., Huang J. C. & Nieh T. G. Inhomogeneous to homogeneous transition in an Au-based metallic glass and its deformation maps. Intermetallics 18, 702–709 (2010).

[b23] Fan Y., Iwashita T. & Egami T. How thermally activated deformation starts in metallic glass. Nat. Commun. 5, 5083 (2014). 25248915 10.1038/ncomms6083

[b24] Lu J., Ravichandran G. & Johnson W. L. Deformation behavior of the Zr_41.2_Ti_13.8_Cu_12.5_Ni_10_Be_22.5_ bulk metallic glass over a wide range of strain-rates and temperatures. Acta Mater. 51, 3429–3443 (2003).

[b25] Wakeda M., Saida J., Li J. & Ogata S. Controlled rejuvenation of amorphous metals with thermal processing. Sci. Rep. 5, 10545 (2015). 26010470 10.1038/srep10545 PMC4443766

[b26] Zhilyaev A. P. & Langdon T. G. Using high-pressure torsion for metal processing: fundamentals and applications. Prog. Mater. Sci. 53, 893–979 (2008).

[b27] Hóbor S. . High pressure torsion of amorphous Cu_60_Zr_30_Ti_10_ alloy. J. Appl. Phys. 104, 033525 (2008).

[b28] Révész Á., Schafler E. & Kovács Z. Structural anisotropy in a Zr_57_Ti_5_Cu_20_Al_10_Ni_8_ bulk metallic glass deformed by high pressure torsion at room temperature. Appl. Phys. Lett. 92, 011910 (2008).

[b29] Dmowski W. . Structural rejuvenation in a bulk metallic glass induced by severe plastic deformation. Acta Mater. 58, 429–438 (2010).

[b30] Meng F., Tsuchiya K., Seiichiro & Yokoyama Y. Reversible transition of deformation mode by structural rejuvenation and relaxation in bulk metallic glass. Appl. Phys. Lett. 101, 121914 (2012).

[b31] Adachi N., Todaka Y., Yokoyama Y. & Umemoto M. Improving the mechanical properties of Zr-based bulk metallic glass by controlling the activation energy for β-relaxation through plastic deformation. Appl. Phys. Lett. 105, 131910 (2014).

[b32] Horstemeyer M. . Torsion/simple shear of single crystal copper. J. Eng. Mater. Technol. 124, 322–328 (2002).

[b33] Zheng B. . Multiple and extended shear band formation in MgCuGd metallic glass during high-pressure torsion. Scripta Mater. 86, 24–27 (2014).

[b34] Shimizu F., Ogata S. & Li J. Theory of shear banding in metallic glasses and molecular dynamics calculations. Mater. Trans. 48, 2923–2927 (2007).

[b35] Cao A., Cheng Y. & Ma E. Structural processes that initiate shear localization in metallic glass. Acta Mater. 57, 5146–5155 (2009).

[b36] Falk M. & Maloney C. Simulating the mechanical response of amorphous solids using atomistic methods. Eur. Phys. J. B 75, 405–413 (2010).

[b37] Homer E. R. Examining the initial stages of shear localization in amorphous metals. Acta Mater. 63, 44–53 (2014).

[b38] Feng S. . Structural feature of Cu_64_Zr_36_ metallic glass on nanoscale: Densely-packed clusters with loosely-packed surroundings. Scripta Mater. 115, 57–61 (2016).

[b39] Jang D. & Greer J. R. Transition from a strong-yet-brittle to a stronger-and-ductile state by size reduction of metallic glasses. Nat. Mater. 9, 215–219 (2010). 20139966 10.1038/nmat2622

[b40] Chen C., Pei Y. & De Hosson J. T. M. Effects of size on the mechanical response of metallic glasses investigated through *in situ* TEM bending and compression experiments. Acta Mater. 58, 189–200 (2010).

[b41] Sha Z.-D. . On the notch sensitivity of CuZr metallic glasses. Appl. Phys. Lett. 103, 081903 (2013).

[b42] Medvedev N. The algorithm for three-dimensional Voronoi polyhedra. J. Comput. Phys. 67, 223–229 (1986).

[b43] Feng S. . Effects of flow defects on hypothetical ZrCu metallic glasses microstructure and plasticity: A molecular dynamics analysis. J. Alloys Compd. 656, 518–523 (2016).

[b44] Honeycutt J. D. & Andersen H. C. Molecular dynamics study of melting and freezing of small Lennard-Jones clusters. J. Phys. Chem. 91, 4950–4963 (1987).

[b45] Pan S. P. . Structural disorder in metallic glass-forming liquids. Sci. Rep. 6, 27708 (2016). 27278113 10.1038/srep27708 PMC4899719

[b46] Manoharan V. N., Elsesser M. T. & Pine D. J. Dense packing and symmetry in small clusters of microspheres. Science 301, 483–487 (2003). 12881563 10.1126/science.1086189

[b47] Trexler M. M. & Thadhani N. N. Mechanical properties of bulk metallic glasses. Prog. Mater. Sci. 55, 759–839 (2010).

[b48] Cheng Y., Cao A. J., Sheng H. & Ma E. Local order influences initiation of plastic flow in metallic glass: Effects of alloy composition and sample cooling history. Acta Mater. 56, 5263–5275 (2008).

[b49] Plimpton S. Fast parallel algorithms for short-range molecular dynamics. J. Comput. Phys. 117, 1–19 (1995).

