# Supplementary material for: Structural evolution of nanoscale metallic glasses during high-pressure torsion: A molecular dynamics analysis
Source: Sci Rep. 2016 Nov 7;6:36627. doi: 10.1038/srep36627 (PMC5098210; doi:10.1038/srep36627)
Supplement: Supplementary Materials [file srep36627-s4.doc]

**Supplementary Materials**

**Structural evolution of** **nanoscale metallic glasses during high-pressure torsion: A** **molecular dynamics analysis**

S.D. Feng^1^, W. Jiao^2^, Q. Jing^1^, L. Qi^1^, S.P. Pan^3^, G. Li^1,4^, M.Z. Ma^1^, W.H. Wang^2^, R.P. Liu^1,*^

*^1^State Key Laboratory of Metastable Materials Science and Technology,* *Yanshan*

*University, Qinhuangdao 066004, China*

*^2^**Institute of Physics,* *Chinese Academy of Sciences, Beijing 100190, China*

*^3^College of Materials Science and Engineering,* *Taiyuan University of Technology, Taiyuan, 030024, China*

*^4^Department of Materials Science and Engineering, The University of Tennessee,*

*Knoxville, Tennessee 37996, USA*

***** Correspondence and requests for materials should be addressed to R.P.L.(email: riping@ysu.edu.cn.)

**Supporting Information**

Molecular dynamics simulation movies of models deformed under high-pressure torsion.

Movie 1: Molecular dynamics simulation of deformation at the outside surface of nanoscale Cu_50_Zr_50_ MGs under high-pressure torsion at 300 K, corresponding to Figure 1 (up).

Movie 2: Molecular dynamics simulation of deformation at cross-section of nanoscale Cu_50_Zr_50_ MGs under high-pressure torsion at 300 K, corresponding to Figure 1 (down).

Movie 3: Molecular dynamics simulation of deformation at the outside surface of nanoscale Cu_50_Zr_50_ crystals under high-pressure torsion at 300 K, corresponding to Figure 2.

**Supplementary Figures**


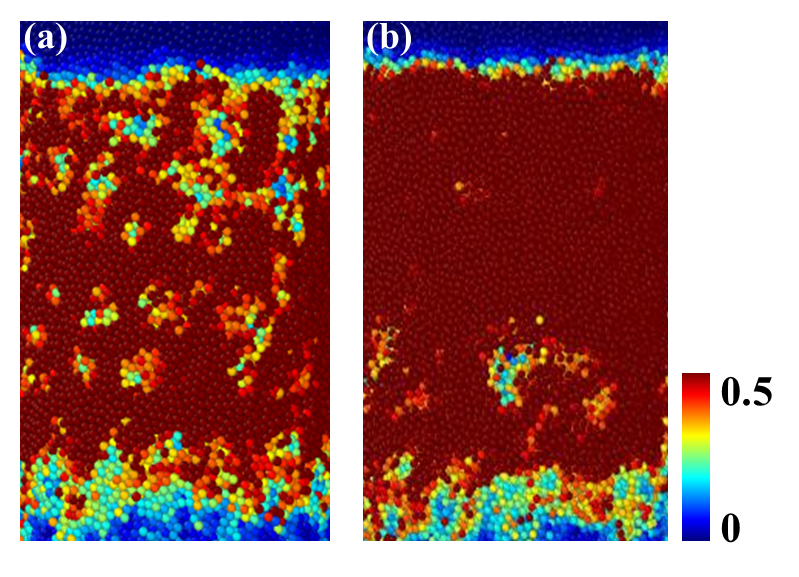


Fig. S1 Local shear strain of models cooled by (a) 1e9 K/s and (b) 5e11 K/s at TA = 180°.

Because of inherent limitations of MD method, the time scale in MD simulation is much shorter than experimental time scale. Notwithstanding, one has to keep in mind that the behavior of materials can be significantly different with different cooling rates. Fig. S1 shows the distribution of atoms with atomic shear strain for different cooling rates. It can be seen that the distribution of shear transformations was affected by cooling rates. With decreasing cooling rates, the shear transformations reduced a little because of no enough free volume allowing this to come about. The effect of cooling rate on the HPT deformation of MGs suggested that a higher cooling rate led to a greater extent of shear transformations. The results indicated that the activation of a high density of shear transformations can readily lead to the suppression of shear bands in MGs. However, in the experiments, the cooling rate is much lower than that in MD simulations. And there is no enough free volume allowing a large number of shear transformations to come about.


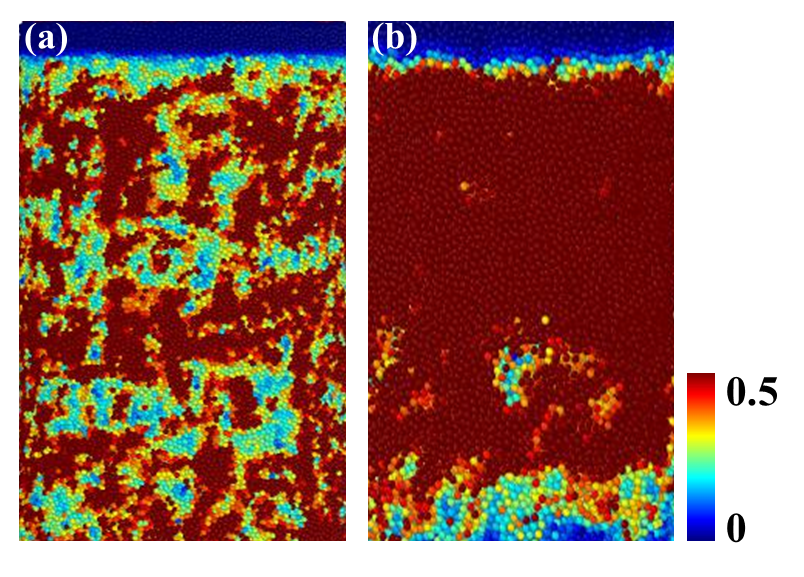


Fig. S2 Local shear strain of models whose diameters are (a) 30 nm and (b) 15 nm at TA = 180°.

We also studied the effects of size on HPT, as shown in Fig. S2. The dimensions of the left cylindrical-shaped nanoscale MG were 30 nm in diameter and 60 nm in the z-axis, while those of the right one were 15 nm in diameter and 30 nm in the z-axis. It can be seen that the accumulation degree of shear transformations was sensitive to the size of models. The percentage of atoms that participate in the shear transformation is decreased with the increase in the size when the TAs were identical. In the small model, almost all atoms were involved in the deformation as shown in Fig. S2(b), while a few parts of atoms were not involved in the shear transformations in the large model. It can be inferred that when the relevant size increases to a critical value, deformation modes change from homogeneous deformation to localized deformation, corresponding to the formation of shear bands. Therefore, the nanoscale MG in MD simulations under HPT deformed homogeneously, leading to the suppression of shear bands in MGs. It is worth noting that, our simulation scale is limited by computer capability at present, and models of beyond nanometer scale cannot be simulated. In the experiment, researchers usually use bulk metallic glasses to study HPT. The size of samples are much bigger than that in MD simulations, so people can find shear bands inside the metallic glass.
